# Development and application of a colloidal-gold immunochromatographic strip for detecting Getah virus antibodies

**DOI:** 10.1007/s00253-024-13168-5

**Published:** 2024-06-01

**Authors:** Zhiwen Jiang, Ying Qin, Letian Zhang, Gang Xing, Zhiyu Shi, Wanjie Song, Georgi M. Dobrikov, Jie Chen, Shuo Su

**Affiliations:** 1 Jiangsu Engineering Laboratory of Animal Immunology, Institute of Immunology and College of Veterinary Medicine, Nanjing Agricultural University, Nanjing, 210095 China; 2 Sanya Institute of Nanjing Agricultural University, Sanya, China; 3 MOA Key Laboratory of Animal Virology, Zhejiang University, Hangzhou, 310058 China; 4 Institute of Organic Chemistry With Centre of Phytochemistry, Bulgarian Academy of Sciences, Acad. G. Bonchev Street, Bl. 9, 1113 Sofia, Bulgaria

**Keywords:** Getah virus, p62-E1 protein, Colloidal gold immunochromatography strip, Rapid antibody detection

## Abstract

**Abstract:**

Getah virus (GETV) is a re-emerging mosquito-borne alphavirus that is highly pathogenic, mainly to pigs and horses. There are no vaccines or treatments available for GETV in swine in China. Therefore, the development of a simple, rapid, specific, and sensitive serological assay for GETV antibodies is essential for the prevention and control of GETV. Current antibody monitoring methods are time-consuming, expensive, and dependent on specialized instrumentation, and these features are not conducive to rapid detection in clinical samples. To address these problem, we developed immunochromatographic test strips (ICTS) using eukaryotically expressed soluble recombinant p62-E1 protein of GETV as a labelled antigen, which has good detection sensitivity and no cross-reactivity with other common porcine virus-positive sera. The ICTS is highly compatible with IFA and ELISA and can be stored for 1 month at 37 °C and for at least 3 months at room temperature. Hence, p62-E1-based ICTS is a rapid, accurate, and convenient method for rapid on-site detection of GETV antibodies.

**Key points:**

*• We established a rapid antibody detection method that can monitor GETV infection*

*• We developed colloidal gold test strips with high sensitivity and specificity*

*• The development of colloidal gold test strips will aid in the field serologic detection of GETV*

**Supplementary Information:**

The online version contains supplementary material available at 10.1007/s00253-024-13168-5.

## Introduction

Getah virus (GETV) is an alphavirus that has re-emerged in the animal industry. It causes abortions in sows, rapid death of newborn piglets, and self-limiting illness in horses, with symptoms including fever and hindlimb enlargement (Lu et al. 2019; Wang et al. 2022b; Yang et al. 2018). GETV has been found in more animals in recent years, including red pandas (Zhao et al. 2022), cattle (Liu et al. 2019), and blue foxes (Shi et al. 2019). In addition, early serologic surveillance has shown the presence of antibodies to GETV in humans, indicating potential infection (Li et al. 2022). However, there is no effective vaccine for controlling GETV epidemics in China, and our preliminary epidemiologic data point to an epidemiologic trend suggested by GETV in recent years (Zhao et al. 2023), making further surveillance and diagnosis of GETV imperative. In addition, the reappearance of GETV in China and even other Asian countries suggests the risk of a continuing epidemic of the virus. In addition to the development of an effective vaccine and higher biosecurity measures, regular surveillance programmes (including antigens or antibodies) are necessary.

GETV is a positive-strand RNA virus belonging to the genus *Alphavirus* of the family *Togaviridae*, whose genome consists of two coding reading frames (ORFs), the first of which encodes nonstructural proteins 1–4 (nsP1-4) and the second of which encodes structural proteins (Cap, E3, E2, 6 k, and E1) (Wang et al. 2022a). The glycoproteins of alphaviruses consist of heterodimers formed by two transmembrane subunits, the E2 and E1 proteins, which mediate viral attachment and membrane fusion, respectively (Zhang et al. 2018). E2 proteins are initially expressed as precursor polypeptides known as p62 proteins. The p62 proteins are cleaved by cellular furin protease to generate the E2 and peripheral E3 polypeptides. The E3 proteins remain bound to the E2/E1 heterodimer, preventing premature conformational changes and membrane fusion (Uchime et al. 2013). The alphavirus E2 and E1 proteins both induce a neutralizing antibody response, making them commonly employed as immunogens or targets for immunological assays. Previous basic studies on other alphaviruses have shown that recombinant p62-E1 heterodimeric proteins expressed by eukaryotic cells are similar in structure to glycoprotein spikes on natural viruses (Voss et al. 2010) and specifically recognize neutralizing antibodies and viral receptors, suggesting that recombinant p62-E1 heterodimeric proteins have great potential as antigens for serological detection of alphaviruses.

Several previous studies have established polymerase chain reaction (PCR) or enzyme-linked immunosorbent assay (ELISA) methods for the detection of GETV (Qiu et al. 2022; Sun et al. 2022; Zhang et al. 2022). However, the rapid dissipation of viremia for alphaviruses makes antigen detection difficult unless the infected piglets have died. ELISA methods for detecting antibody IgG are mostly based on the E2 protein, the key glycoprotein encoded by GETV, which is responsible for receptor binding and rich in antigenic epitopes. They were all developed based on prokaryotic expression systems that require protein dialysis, and inclusion body proteins do not readily maintain the basic conformation of the protein. Furthermore, further optimization of antigenic targets is necessary. Viral neutralization assays and ELISA are used to detect antibodies with high sensitivity and specificity, but they mostly rely on specialized instrumentation and laboratory techniques, which tends to limit their use for rapid detection in the clinical setting. With the prevalence of severe acute respiratory syndrome coronavirus 2 (SARS-CoV-2) in recent years, lateral flow assays (LFAs) have been more frequently established for the rapid detection of antigens or antibodies (Cavalera et al. 2021), and this method has provided a reliable means of improving detection requirements and making diagnoses in remote areas. Accordingly, colloidal gold immunochromatographic test strips are capable of rapid detection of antigens or antibodies; they are relatively inexpensive and easy to operate, have good specificity and sensitivity, and have been used to detect antibodies to many viruses.

In this study, we established a test strip capable of rapid detection of GETV antibodies based on colloidal gold immunochromatographic test strip technology, which is based on GETV recombinant p62-E1 protein with high sensitivity and good specificity. Moreover, the test strip is expected to be used for preliminary serological screening of GETV to better assess the natural infection of GETV in livestock and poultry. With the development of commercial vaccines, it is also possible to evaluate the immunization effect of the vaccine and the level of maternal antibodies in piglets.

## Materials and methods

### Reagents and instruments

Staphylococcal protein A (SPA), FITC-labelled rabbit anti-pig IgG, HRP-labelled rabbit anti-pig IgG, and HRP-labelled goat anti-mouse IgG were purchased from Solarbio (Beijing, China). Bovine serum albumin (BSA) was purchased from Sigma‒Aldrich (St. Louis, MO, USA). Strep-Tactin XT 4Flow high-capacity resin was purchased from IBA Lifesciences (Germany). SMM 293-TII Expression Medium and SMS 293-SUPI Expression Medium Supplement were purchased from Sino Biological (Beijing, China).

Colloidal gold (30 nm), nitrocellulose (NC) membranes, glass fibre filter membranes, absorbent pads, and PVC substrate were purchased from Shanghai JieYi Biotechnology Co., Ltd., China. Mouse anti-GETV E1 monoclonal antibody (4D10) was prepared and stored in our laboratory. All other reagents and solvents were of analytical grade or higher quality. The XYZ large platform 3D induction scribing and gold spraying instrument HM3260 and dual correction numerical control slitting machine SPT300 were purchased from Shanghai Gold Biotechnology Co., Ltd.

### Expression and purification of soluble GETV p62-E1 protein

**Fig. 1:**
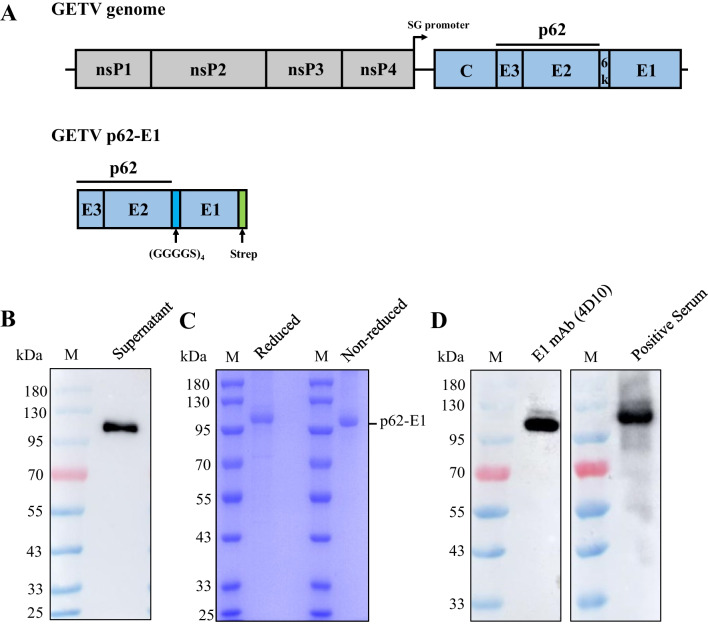
Expression and purification identification of recombinant p62-E1 protein in the Expi293F system. **A** Schematic of the GETV genome and p62-E1 construct. **B** Western blot detection of p62-E1 protein after transfection. **C** SDS‒PAGE analysis of purified p62-E1 protein. **D** Western blot analysis of purified recombinant p62-E1 protein

A mammalian cell codon-optimized GETV full-length E gene plasmid (the sequence is included in the supplemental material) was prepared in previous studies (Zhai et al., unpublished). Then, we designed forwards and reverse primers for the target gene and performed PCR to separately amplify the GETV-p62 (E3 and E2) and GETV-E1 genes, removing their transmembrane region (TM). The p62 and E1 genes were then fused using overlap PCR, with a homologous arm containing the linker sequence (GGGGS)_4_ between the two genes (Fig. 1A). After digestion with *Xba*I and *Kpn*I enzymes, the fused fragment was ligated to the pcDNA3.4 plasmid to construct the expression vector. There are Strep and 6 × His tag sequences on the C-terminal of the target protein, which can be used for protein purification.

Expi293F cells were transfected with the recombinant expression plasmid. The cell density was adjusted to 2.5 × 10^6^ cells/mL. After 24 h of transfection, 3.5 mL of SMS 293-SUPI Expression Medium Supplement (Sino Biological, Beijing, China) was added per 0.1 L of culture. The cells were incubated in the culture incubator for 4 days to allow protein expression. After the incubation period, the supernatant was harvested for protein purification. Protein purification was performed using Strep-Tactin XT 4Flow high-capacity resin (IBA Lifesciences, Germany) according to the manufacturer’s instructions. After protein purification, the purified protein was concentrated, and the buffer was exchanged with PBS (pH = 7.4). The protein concentration was determined using the Bradford assay. The protein was divided into aliquots and stored at − 80 °C for future use.

The purified p62-E1 was analyzed by sodium dodecyl sulfate–polyacrylamide gel electrophoresis (SDS‒PAGE). The antigenicity of the purified p62-E1 was tested by Western blot (WB) using GETV-positive serum or E1-4D10 mAb. Specifically, p62-E1 was separated by SDS-PAGE and then transferred to NC membranes. Membranes were blocked with 5% skim milk in PBST (containing 0.1% Tween-20) for 2 h at room temperature (RT) and then incubated with GETV-positive serum (1:500 dilution) or E1-4D10 mAb (1:2000 dilution) overnight at 4 °C. After washing five times with PBST, the membranes were treated with HRP-labelled rabbit anti-pig IgG or HRP-labelled goat anti-mouse IgG (1:10,000; Solarbio, Beijing, China) for 1 h at room temperature. After washing the membrane five times with PBST, the protein was visualized using an Amersham Imager 600.

### Preparation of recombinant p62-E1 protein-based colloidal gold solution

The methodology for determining the optimal antigen amount and labelling conditions was based on a previous report (Bai et al. 2022). After that, the colloidal gold solution pH was adjusted to the optimal pH by adding 8 μL of 0.2 M K_2_CO_3_ per mL colloidal gold solution. After adding the optimal protein labelling concentration of 8 μg/mL recombinant p62-E1 protein, labelling was performed in a glass beaker that had been treated with acid. During the process, the mixture was stirred to ensure thorough labelling. After 30 min of labelling, 100 μL of 10% bovine serum albumin (BSA) blocking solution (final concentration of 1%) was added for each millilitre of colloidal gold solution to block any unbound colloidal gold.

**Fig. 2:**
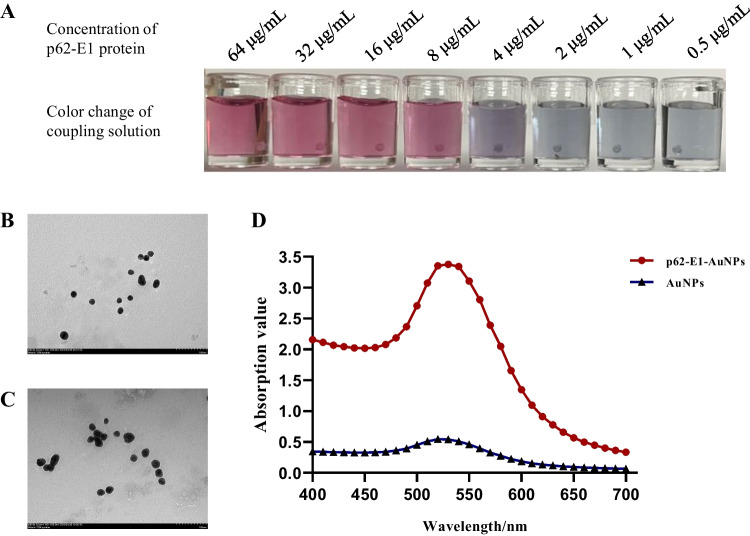
Optimization of the colloidal gold solution. **A** Determination of the optimal protein concentration for conjugation. The colour of the mixture changed from red to deep purple. The optimal protein concentration for colloidal gold labelling was determined to be 8 μg/mL. **B-C** Transmission electron microscopy (TEM) analysis of AuNPs (**B**) and p62-E1-AuNPs (**C**). **D** UV scanning peaks. The maximum absorption peak of p62-E1-AuNPs was 530 nm. AuNPs, gold nanoparticles; p62-E1-AuNPs, colloidal gold-labelled p62-E1

After labelling, the labelled complex was centrifuged at 2000 rpm and 4 °C for 5 min to remove any unbound colloidal gold particles. The supernatant was collected carefully and then centrifuged at 12,000 rpm and 4 °C for 30 min. The supernatant was removed, and the pellet was resuspended in the gold standard protein resuspension buffer (0.02 M borate buffer, pH = 9.0, 0.1% casein, 0.2% PEG20000). The volume of the resuspension buffer was 1/10 of the original liquid volume. After resuspension, the solution was stored in a light-protected container at 4 °C for future use. Using transmission electron microscopy (TEM), the uniformity and morphology of the gold-labelled p62-E1 protein particles were analysed (Fig. 2).

### Assembly of recombinant p62-E1 protein-based ICTS

Rapid diagnostic test strips (p62-E1-based ICTSs) were constructed using three pads: an absorbent pad measuring 30 by 1.8 cm, a conjugate pad measuring 30 by 0.6 cm, and a sample pad measuring 30 by 1.8 cm. These pads were assembled along with an NC membrane measuring 30 by 1.5 cm onto a PVC substrate. The gold pad was immersed in the gold pad processing solution (0.02 M borate buffer, pH = 9.0, 2% sucrose, 2% trehalose, 0.1% Triton X-100, 0.1% PVP-10) for 10 min. After removal, it was dried at 37 °C for 2 h. The gold-labelled protein was sprayed onto the gold pad using a spraying instrument at a rate of 8 μL/cm. The gold pad was then placed in a vacuum drying oven and dried at 37 °C for 1 h. SPA and mouse anti-E1 mAb 4D10 were diluted in 0.01 M Tris buffer (pH = 8.5) to final concentrations of 0.5 mg/mL and 1.3 mg/mL, respectively. Both solutions were sprayed onto nitrocellulose membranes at a rate of 1 μL/cm to form the test line (T-line) and the control line (C-line). The sample pad was immersed in the sample pad processing solution (0.02 M borate buffer, pH = 9.0, containing 0.5% Tween-20, 2% PEG20000, 1% BSA, 0.1% PVP-10) for 10 min and then dried at 37 °C for 2 h. The NC membrane, gold pad, sample pad, and absorbent pad were assembled in sequence, adhering them to the PVC substrate. Then, the assembled layers were cut into colloidal gold test strips with a width of 0.3 cm. Finally, the test strips were placed into the card housing for packaging and vacuum sealing in plastic bags containing desiccants.

### Principle and result readout

**Fig. 3:**
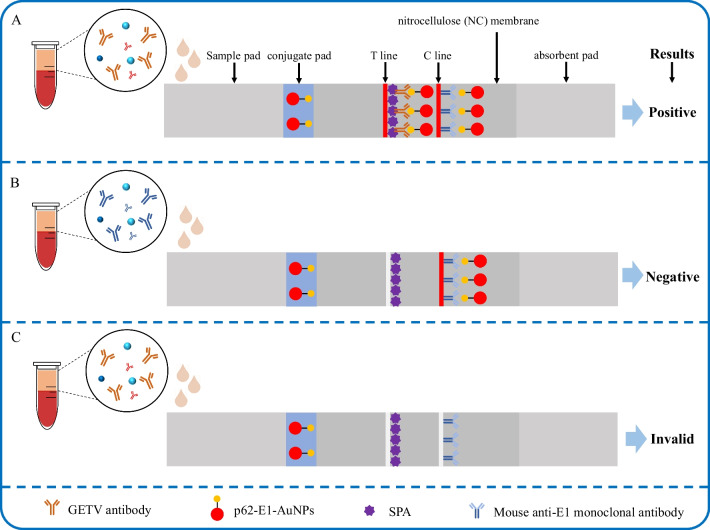
Schematic diagram and results analysis of p62-E1-based ICTSs. **A** For GETV-positive serum samples, the gold-labelled p62-E1 protein forms a complex with anti-p62-E1 protein IgG before binding with SPA. SPA binds to the Fc region of the antigen–antibody complex, causing the T line with SPA to turn red. **B** For GETV-negative serum samples, the gold-labelled p62-E1 protein only forms a complex with the E1 monoclonal antibody on the C line, resulting in a red colour on the C line and no colour on the T line. **C** If the test strip is ineffective or improperly operated, the gold-labelled protein will not bind to the C line and T line. As a result, both the C line and T line will lack colour

Drops of 100 μL of serum diluted (1:100) in the sample diluent were added to the sample well of the ICTS. Through capillary action, the antibodies in the serum and the colloidal gold-labelled protein were carried along by the sample serum, diffusing towards the nitrocellulose membrane and eventually reaching the absorbent pad at the handle end. During the diffusion process, antibodies in the sample serum bound to the gold-labelled protein, forming reddish-brown marks where they bound to the SPA on the test line (T-line) of the nitrocellulose membrane. The gold-labelled protein also bound to the anti-E1 mAb on the control line (C-line), resulting in a reddish-brown mark. If there were no GETV antibodies in the serum, only a single reddish-brown mark would appear at the control line. If there were GETV-specific antibodies in the serum, reddish-brown marks would appear at both the control and test lines. Conversely, the absence of any marks on the test strip indicated either a faulty test strip or an operational error (Fig. 3).

### Performance testing of ICTS based on recombinant p62-E1 protein

To evaluate the performance of the ICTS developed using p62-E1 protein, a series of experiments were conducted to assess their specificity, sensitivity, and stability. Following the aforementioned detection method, the serum samples were diluted at 1:100 and added to the sample well, and the results were determined within 5–15 min.

First, the test strips were used to detect sera positive for CSFV, PRRSV, PRV, JEV, and PCV2 to evaluate the specificity of the test strip. Second, the serum samples positive for GETV were serially diluted from 1:100 to 1:51,200 in twofold increments to assess sensitivity. To assess the stability of the test strips, the specificity and sensitivity were evaluated at room temperature and 37 °C under vacuum-sealed conditions at intervals of 0, 1, 2, and 3 months after preparation. Repeatability experiments were conducted by separately labelling colloidal gold with proteins from different batches (labelled as 2023.4.20, 2023.5.3, and 2023.5.20), assembling them into test strips, and then testing their sensitivity and specificity.

### Determination of antibody titres using indirect ELISA

To determine the sensitivity and accuracy of colloidal gold test strips, we developed an indirect ELISA based on the p62-E1 protein to detect GETV serum antibody titres. The ELISA was optimized using a checkerboard method (Qiu et al. 2022), and subsequent experiments were performed according to the optimal protocol. Briefly, each well of a 96-well microplate was coated with 100 μL of 0.5 μg/mL recombinant GETV p62-E1 protein and incubated overnight at 4 °C. The plate was washed five times with PBST. After blocking with 5% skim milk for 1 h at 37 °C, the plate was washed five times with PBST and then incubated with the test serum at a 1:100 dilution ratio at 37 °C for 1 h. After five washes, the plate was incubated at 37 °C for 45 min with HRP-labelled rabbit anti-pig IgG diluted 1:10,000 in PBST. Finally, the plate was washed five times, and 100 μL of TMB chromogenic substrate was added and incubated at 37 °C for 15 min. The reaction was stopped by adding 50 μL of sulfuric acid stop solution. The absorbance at 450 nm was measured using a microplate reader.

### Indirect immunofluorescence assay

To confirm the accuracy of the experiment, indirect immunofluorescence assay (IFA) was employed as a supplementary validation method for ICTS and ELISA. Each well of a 96-well microplate was infected with GETV (GETV-HN: MZ736801) at MOI = 1 and incubated at 37 °C for 12 h. Then, the cells were fixed with paraformaldehyde solution and air-dried. Permeabilize with 0.1% Triton X-100 for 15 min, followed by five PBST washes. The membrane was blocked with 5% skim milk for 1 h at 37 °C. The cells were incubated with serum diluted in PBS at a ratio of 1:500 at RT for 2 h, followed by five washes with PBST. Then, fluorescein isothiocyanate (FITC)–labelled rabbit anti-pig IgG (Solarbio, Beijing, China) diluted in PBS was added (100 μL) at a ratio of 1:500 and incubated at RT for 1 h, followed by five PBST washes. Finally, 4′,6-diamidino-2-phenylindole (DAPI) was diluted at a ratio of 1:500, and 100 μL was added per well for nuclear staining. The fluorescence patterns were observed under a fluorescence microscope.

### Detection of GETV Abs in clinical serum samples

The accuracy of the test strip was evaluated using 69 clinical serum samples through the application of the ICTS based on recombinant p62-E1 protein and the established ELISA method. Collected pig serum samples were diluted at a 1:100 ratio and added to the developed test strip for GETV antibody detection. The results were read out within 15 min. Each serum sample was tested in duplicate, and simultaneously, the same serum samples were subjected to ELISA analysis. The IFA method was employed as an additional assessment. The coincidence rate of the GETV antibody colloidal gold test strip was compared with that of the ELISA and IFA tests.

## Results

### Expression and purification of GETV p62-E1 protein

The construction of p62-E1 is shown in Fig. 1A. Recombinant p62-E1 protein expression and purification in Expi293F cells were analyzed using SDS‒PAGE and Western blotting. Western blot analysis of the cell culture medium at 3 days post-transfection to assess protein expression showed that the plasmid could be secreted and expressed in Expi293F cells (Fig. 1B). Reduced and nonreduced SDS‒PAGE analysis after purification showed distinct bands at approximately 100 kDa, suggesting that the p62-E1 protein exists in a heterodimeric form and that the purity was high (Fig. 1C). Western blot analysis indicated that p62-E1 protein exhibited strong reactivity and specificity with mouse anti-E1 monoclonal antibody and positive porcine GETV serum (Fig. 1D). The protein concentration was determined by the Bradford method and adjusted to 1 mg/mL, and the samples were stored at − 80 °C.

### Optimization results of the colloidal gold-based p62-E1 ICTS system

By adding varying amounts of 0.2 mol/L K_2_CO_3_ to adjust the pH of the colloidal gold solution, the optimal labelling pH was determined to be between 7.0 and 7.5, matching the addition of 8 µL K_2_CO_3_. The optimization process for labelling concentration is described below. Ten microlitres of p62-E1 protein with final concentrations of 0.5, 1, 2, 4, 8, 16, and 32 μg/mL was used to label 125 μL of colloidal gold, and then 125 μL of 10% sodium chloride solution was added. The plate was left at room temperature for 5 min to observe the colour change of the solution. The protein concentration at which no colour change occurred was considered the optimal protein concentration for stabilizing the colloidal gold (Fig. 2A). The colour of the mixture changed from red to deep purple. Thus, the optimal protein concentration for labelling was determined to be 8 μg/mL. After conjugation, the structure of the gold-labelled protein particles was observed using electron microscopy (Fig. 2B). Compared to unmodified colloidal gold, the gold-labelled protein particles exhibited a significant increase in particle size, indicating successful labelling (Fig. 2C). The optimal conditions for the ICTS are presented in Table 1.

**Table 1:** The optimal conditions for the ICTSs

Condition	Result
Optimal labelling pH	7.0–7.5
Concentration of labelled protein	8 μg/mL
Concentration of SPA	0.5 mg/mL
Anti-E1 mAbs concentration	1.3 mg/mL
Volume of sprayed p62-E1-gold protein	8 µL/cm
T-line and C-line dilution solution	10 mM Tris (pH = 8.5)

### Specificity of p62-E1-based ICTS

**Fig. 4:**
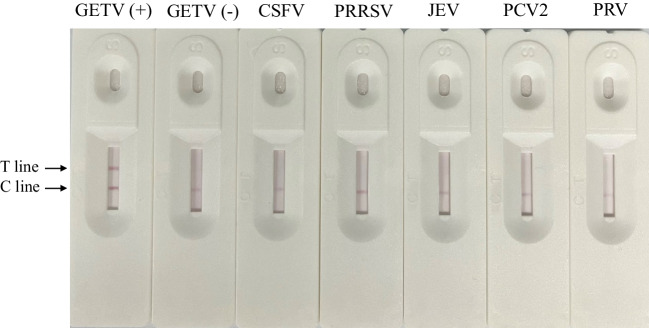
The specificity of p62-E1-based ICTSs. To evaluate the specificity of the test strips, six different types of pig serum samples positive for GETV, CSFV, PRRSV, JEV, PRV, and PCV2 were used for testing. C, control line; T, test line. The GETV-positive serum displayed a clear C line and T line, while other sera only showed a clear C-line. No cross-reactivity was observed, indicating that this ICTS is specific for GETV-positive serum samples

To assess the specificity of the test strips, sera positive for CSFV, PRRSV, PRV, JEV, and PCV2 were tested using the developed test strips (Fig. 4). When diluted at 1:100, all test strips exhibited a clear C-line, confirming the effectiveness of the test. The GETV-positive serum showed a distinct T-line, while the T-lines for the other sera remained absent, indicating the absence of cross-reactivity and affirming the high specificity of the test strip.

### Sensitivity, stability, and repeatability of p62-E1-based ICTS

**Fig. 5:**
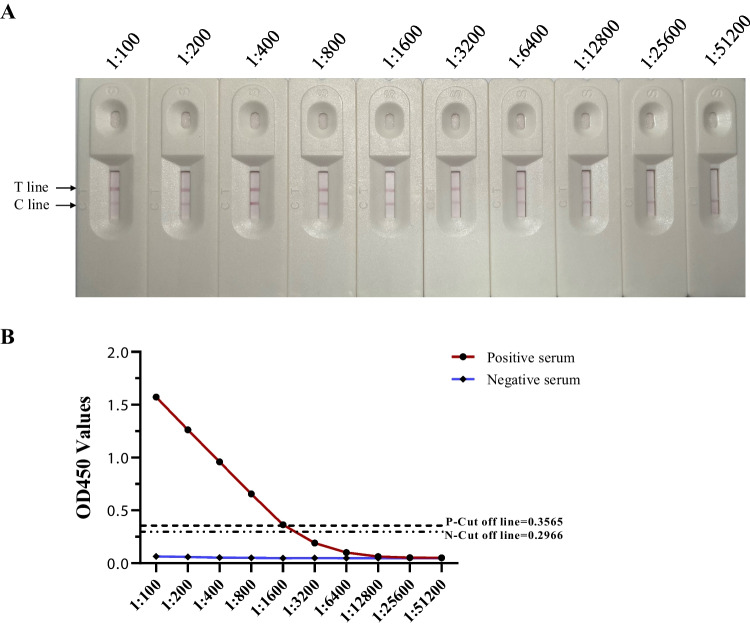
The sensitivity of p62-E1-based ICTSs. **A** GETV-positive serum was serially diluted starting from 1:100 and diluted by twofold up to 1:51,200 to test the sensitivity of the ICTS. C, control line; T, test line. A clear T-line was observed even at a dilution of 1:12,800, while at a dilution of 1:25,600, the T-line was faint and almost invisible. **B** Sensitivity determination of p62-E1 based ELISA

To evaluate the sensitivity of the test strip for GETV, the positive serum was initially diluted at 1:100 and subsequently subjected to consecutive twofold dilutions, with the final dilution reaching 1:51,200. As depicted in Fig. 5A, the test strip still displayed a visible T-line at serum dilutions of up to 1:12,800. At a serum dilution of 1:25,600, the T-line became faint and nearly imperceptible to the naked eye. When the serum dilution reached 1:51,200, no bands appeared on the T-line. Hence, we suggest that the detection limit of the test strip was determined to be 1:12,800 (Fig. 5A). As a control, we conducted ELISA testing with different dilutions and measured OD450 values. We found that at a dilution of 1:1600, ELISA was determined to be positive, and wells larger than this dilution were determined to be negative. At a dilution of 1:12,800, the reading was approximately equivalent to that of the blank control (Fig. 5B). This demonstrates a strong correlation between ICTS and ELISA methods, with ICTS showing higher sensitivity than the ELISA method.

Stability is an important factor in field applications. To assess the stability of the test strip, its sensitivity for detecting positive serum samples was evaluated at different time points, after storage at room temperature and at 37 °C. The results are summarized in Table 2. The p62-E1-based test strip maintained its sensitivity for at least 3 months at room temperature and at least 1 month at 37 °C, indicating its robust stability over an extended period. Finally, we conducted tests using three batches of p62-E1 protein prepared at different times (labelled 2023.4.20, 2023.5.3, and 2023.5.20) to assess the repeatability of the colloidal gold test strips. The results showed no differences in sensitivity and specificity among the three batches. This indicates that the ICTS exhibits excellent repeatability.

**Table 2:** The stability of the p62-E1-based ICTSs

Temperature	Sensitivity over storage time (months)			
	0	1	2	3
18–25 °C (RT^a^)	1: 12,800	1:12,800	1:12,800	1:12,800
37 ℃		1:12,800	ND^b^	ND^b^

^a^Room temperature (RT)

^b^Sensitivity was evaluated for a period of only one month under 37 °C storage. No detection (ND)

### Accuracy of p62-E1-based ICTS for detection of clinical serum samples

Simultaneous testing of 69 clinical serum samples using our established ELISA and test strips was prepared in this study. Additionally, IFA was utilized as an auxiliary verification method to further confirm the accuracy of the results. The positive serum samples exhibited bright green fluorescence when observed under a fluorescence microscope, while the negative control showed no fluorescence. This confirmed the feasibility of using the IFA method to determine the seropositivity of the serum samples (Cao et al. 2023). The findings revealed that 39.13% (27/69) of the samples were positive, which matched the positive samples detected by both ELISA and IFA (Figure S1) methods. The positive coincidence rate between the test strip and the ELISA tests was 100% (27/27), and the positive coincidence rate between the test strip and the IFA tests was also 100% (27/27) (Table 3). Correspondingly, the negative coincidence rate of the three methods was also 100%.

**Table 3:** Concordance rate of the three detection methods

Method		p62-E1-based ICTSs	
		Positive	Negative
ELISA	Positive	27	0
	Negative	0	42
	Positive/negative rates	27/69 (39.13%)	42/69 (60.87%)
Diagnostic coincidence rate		100%	100%
IFA	Positive	27	0
	Negative	0	42
	Positive/negative rates	27/69 (39.13%)	42/69 (60.87%)
Diagnostic coincidence rate		100%	100%

The colloidal gold test strip based on p62-E1 demonstrated good consistency with the established ELISA and IFA methods. Moreover, ICTSs are more convenient and rapid, as they do not require sophisticated laboratory conditions, can be employed to test serum samples even in resource-limited settings such as grassroots breeding farms, and have good specificity and sensitivity.

## Discussion

In China, GETV is a re-emerging virus in pigs that primarily affects the health of newborn piglets and pregnant sows (Yang et al. 2018). Our previous studies have shown that the prevalence of GETV is on the rise, and in the absence of a commercial vaccine, early, effective, and convenient detection as well as the development of scientific preventive and control measures are crucial for the prevention and control of GETV. Hence, there is an urgent need to establish an efficient and convenient method for GETV antibody monitoring.

In addition to IFA and WB, a number of antibody ELISAs have been established based on recombinant E2 proteins with high sensitivity for rapid detection of GETV antibodies in serum (Qiu et al. 2022). However, these methods need specialized experimental instruments or highly skilled assayers to avoid potential operator differences. Compared with these methods, the colloidal gold immunochromatographic detection technique is simple, exhibits rapid response, and does not depend on instrumentation (Li et al. 2023). After diluting the serum to be examined, the experimental results can be observed within 5–15 min, and the technique is more suitable for on-site testing on farms. The target antigen used in this study was the p62-E1 protein, which is derived from a eukaryotic expression system and ensures protein modification. Importantly, in addition to E2, the recombinant E1 protein is equally capable of producing neutralizing or nonneutralizing antibodies (Kim et al. 2021; Williamson et al. 2021), indicating that using p62-E1 heterodimer protein could be able to further improve the sensitivity and specificity of colloidal gold test strips. Compared with the traditional immunoassay methods, the preparation of colloidal gold test strip materials, such as colloidal gold, glass fibre, and absorbent paper, has the characteristics of low cost. The preparation process is also relatively simple and allows for storage at room temperature. Furthermore, the concentration of the colloidal gold-labeled protein was only 8 μg/mL, and utilizing the highly expressed mammalian cell preparation of the protein further decreased the unit cost. Consequently, colloidal gold strips present a more economically viable option for clinical applications.

The quality of p62-E1-based colloidal gold test strips is particularly important in field applications. The results showed that the ICTS had good specificity for detection of GETV, CSFV, PRRSV, JEV, PRV, and PCV2 positive sera. In addition, dilutions using GETV-positive sera to assess the sensitivity of the test strips showed that the test strips had high sensitivity compared to ELISA. Importantly, the test strip results were highly similar to those of IFA and ELISA, indicating that the p62-E1-based test strips can rapidly and accurately detect GETV antibodies. Moreover, considering that SPA can bind IgG from multiple species and subtypes, our test strips can not only detect GETV antibody levels in pigs of all ages (including maternal antibody levels in neonatal piglets) but also monitor GETV antibodies in other species, including animals such as horses and cattle, and even humans (Li et al. 2017).

The prokaryotic expression of E2 protein requires a series of operations, such as dialysis, and does not guarantee the correct folding of the protein, which undoubtedly impairs the sensitivity and specificity of the detection reagents. Our established GETV antibody colloidal gold is the first report based on recombinant p62-E1 protein as a target antigen, and the soluble expression of the p62-E1 protein ensures the true conformation of the alphavirus glycoprotein, as expressed in previous studies. Considering the high relevance of the alphavirus glycoprotein (Kim and Diamond 2023), a similar strategy could be adopted to establish colloidal gold test strips for the rapid antibody detection of other highly pathogenic human alphaviruses (e.g., chikungunya virus (CHIKV) and ross tiver virus (RRV)) (Malonis et al. 2021), which would facilitate antibody surveillance in remote areas. It is important to note that high antigenic correlation of the same serogroup of alphavirus may result in false-positive results (as in the case of CHIKV and O’nyong’nyong virus (ONNV), they are highly antigenically correlated), and further confirmation of the diagnosis in areas where multiple alphaviruses are endemic at the same time may require follow-up with specific laboratory diagnostics (Martins et al. 2019; Nguyen et al. 2020).

In conclusion, colloidal gold test strips based on recombinant p62-E1 antigen can be used as an effective means for rapid clinical detection of GETV antibodies. This approach is low cost (8 μg of p62-E1 protein produces about 40 strips), easy to operate, and suitable for the detection of natural GETV infections in swine herds by breeders. In addition, with continued research, an effective GETV vaccine can be developed in the future, and pigs can quickly produce long-term and effective GETV antibodies after immunization. Our test strip can be used to detect the immunization effect of the vaccine, as well as the screening of maternal antibodies in newborn piglets, which will be conducive to the scientific prevention and control of GETV in the future.

## Supplementary Information

Below is the link to the electronic supplementary material.

Supplementary file1 (PDF 1934 KB)

## Data Availability

The authors can confirm that all relevant data are included in the article and/or its supplementary information files.
