# Supplementary material for: Development and application of a colloidal-gold immunochromatographic strip for detecting Getah virus antibodies
Source: Appl Microbiol Biotechnol. 2024 Jun 1;108:355. doi: 10.1007/s00253-024-13168-5 (PMC11144135; doi:10.1007/s00253-024-13168-5)
Supplement: Supplementary file 1 [file 253_2024_13168_MOESM1_ESM.pdf]

Journal name：Applied Microbiology and Biotechnology

Development and application of a colloidal-gold immunochromatographic strip for detecting Getah virus antibodies

Zhiwen Jiang[1,2 #], Ying Qin[1,2 #], Letian Zhang[1], Gang Xing[3], Zhiyu Shi[1], Wanjie Song[1], Georgi Dobrikov[4], Jie Chen[1] and Shuo Su[1,2 *]

1 Jiangsu Engineering Laboratory of Animal Immunology, Institute of Immunology and College of Veterinary Medicine, Nanjing Agricultural University, Nanjing 210095, China

2 Sanya Institute of Nanjing Agricultural University, Sanya, China

3 MOA Key Laboratory of Animal Virology, Zhejiang University, Hangzhou 310058, China

4 Institute of Organic Chemistry with Centre of Phytochemistry, Bulgarian Academy of Sciences, Acad. G. Bonchev Street, Bl. 9, 1113 Sofia, Bulgaria

* Corresponding author: E-mail addresses: shuosu@njau.edu.cn.

[#] These authors are contributed equally to this work.

The sequence of mammalian cell codon-optimized GETV full-length E gene plasmid：

TCCGCCGCCCTGATGATGTGCGTGCTGGCCAATGTGACCTTCCCCTGTAGCGAGCCCGCCTGCGCCCCCTGCTGCTATGAAAAGCAGCCTGAGC
AGACCCTGAGGATGCTGGAGGACAATGTGGATAGACCCGGCTACTACGACCTGCTGGAGGCCACCATGACTTGTAATAACTCTGCCAGGCACC
GCCGGAGCGTGACCGAGCACTTTAATGTGTACAAGGCCACAAAGCCCTATCTGGCCTACTGTGCCGATTGCGGCGACGGCCAGTTCTGTTACTC
CCCCGTGGCTATTGAGAAGATCCGCGATGAGGCCTCCGACGGCATGATCAAGATCCAGGTGGCCGCCCAGATCGGAATTAACAAAGGCGGCAC
CCACGAGCACAATAAGATCAGGTACATCGCCGGGCATGACATGAAAGAGGCCAACCGGGATAGCCTGCAGGTGCACACCAGCGGGGTCTGCG
CTATCCGGGGAACCATGGGCCATTTCATCGTGGCCTATTGCCCTCCAGGCGACGAGCTGAAAGTGCAGTTCCAGGACGCCGAAAGTCACACCC
AGGCTTGTAAGGTGCAGTACAAGCACGCCCCTGCCCCCGTGGGGAGGGAGAAGTTCACTGTGAGGCCCCATTTTGGCATCGAAGTGCCCTGTA
CCACATACCAGCTGACCACAGCCCCCACCGAGGAAGAGATCGATATGCACACACCTCCCGACATCCCAGACATCACCCTGCTGAGCCAGCAGT
CAGGCAACGTGAAGATCACAGCCGGCGGAAAGACAATTAGATATAACTGCACCTGCGGCAGCGGGAACGTGGGCACCACCTCCTCCGATAAA
ACAATCAACTCCTGTAAGATTGCCCAGTGCCACGCCGCCGTGACCAACCATGACAAGTGGCAGTATACCTCTTCTTTCGTGCCACGGGCTGATC
AGCTGAGCAGAAAGGGCAAGGTGCACGTGCCATTTCCCCTGACCAATTCCACCTGCCGCGTCCCTGTGGCTAGAGCCCCTGGCGTGACCTACG
GCAAACGCGAACTGACCGTGAAGCTGCACCCAGATCACCCAACCCTGCTGACCTATCGGTCTCTGGGCGCCGACCCCCGGCCTTATGAGGAGT
GGATCGACCGGTACGTGGAGCGAACTATTCCCGTGACAGAGGATGGGATTGAATACCGCTGGGGCAACAACCCACCTGTGAGACTGTGGGCAC
AGCTGACCACAGAGGGAAAGCCTCACGGGTGGCCCCACGAGATCATCCTGTATTACTACGGGCTGTATCCTGCCGCCACCATCGCCGCCGTGA
GCGCCGCCGGCCTGGCCGTGGTGCTGAGCCTGCTGGCCAGCTGCTATATGTTCGCCACCGCCAGACGGAAATGTCTGACACCCTACGCACTGA
CCCCCGGAGCCGTGGTCCCCGTGACACTGGGCGTGCTGTGTTGCGCCCCCAGGGCCCACGCCGCTAGCTTTGCAGAGTCTATGGCATACCTGTG
GGATGAGAACCAGACCCTGTTCTGGCTGGAGCTGGCCACCCCCCTGGCCGCTATCATTATCCTGGTGTGCTGCCTGAAAAACCTGCTGTGCTGC
TGTAAGCCCCTGAGCTTTCTTGTGCTGGTGAGCCTGGGAACCCCAGTGGTGAAGTCCTATGAGCACACCGCTACAATCCCCAACGTGGTGGGC
TTCCCCTATAAGGCTCATATCGAGCGGAATGGGTTCAGCCCCATGACCCTGCAGCTGGAAGTGCTGGGCACTAGCCTGGAGCCCACTCTGAACC
TGGAGTACATTACATGCGAGTATAAGACCGTGGTGCCCTCCCCATACATCAAGTGTTGTGGCACATCCGAGTGCAGGAGCATGGAGCGCCCCGA
CTATCAGTGCCAGGTGTACACAGGCGTGTATCCTTTCATGTGGGGCGGGGCCTATTGTTTTTGTGACACCGAGAATACCCAGCTCTCCGAGGCCT
ACGTGGATCGGTCTGACGTGTGCAAGCACGACCACGCTGCCGCCTACAAGGCCCACACCGCCGCCATGAAGGCCACCATTCGCATTTCCTACG
GCAACCTGAATCAGACCACCACAGCCTTCGTGAACGGCGAGCATACCGTGACCGTGGGCGGATCCCGGTTCACCTTCGGCCCAATTTCTACCG
CCTGGACCCCATTTGACAACAAGATTGTGGTGTATAAGAACGACGTGTACAATCAGGACTTCCCTCCCTACGGCAGCGGGCAGCCCGGCAGGT

TCGGGGACATCCAGTCCCGGACCGTGGAGTCCAAGGACCTGTACGCCAACACCGCCCTGAAGCTGAGCCGGCCATCTTCAGGAACAGTGCAC
GTGCCCTATACCCAGACCCCTAGCGGCTTCAAGTACTGGATCAAGGAGCGGGGCACAAGCCTGAATGATAAGGCCCCATTCGGGTGCGTGATCA
AGACCAACCCCGTGCGCGCCGAGAATTGCGCCGTGGGCAATATCCCCGTGAGCATGGACATTCCCGATACCGCCTTCACCAGAGTGATCGATGC
CCCCGCCGTGACAAACCTGGAGTGCCAGGTGGCCGTGTGCACTCACTCCAGCGACTTTGGAGGAATTGCCACCCTGACTTTCAAGACCGATAA
GCCTGGAAAGTGCGCCGTGCACTCCCACTCTAACGTGGCCACCATTCAGGAGGCCGCCGTGGACATTAAGACCGACGGCAAGATCACCCTGCA
CTTCAGCACCGCCTCCGCCAGCCCCGCCTTCAAGGTGAGCGTGTGTAGCGCCAAGACCACCTGCATGGCCGCCTGTGAGCCTCCTAAGGATCA
CATCGTGCCCTACGGCGCCTCTCACAATAATCAGGTGTTTCCTGACATGAGCGGCACCGCCATGACCTGGGTGCAGAGAGTGGCCGGCGGCCT
GGGCGGCCTGACCCTGGCCGCCGTGGCCGTGCTGATCCTGGTGACCTGCGTGACCATGAGGAGA

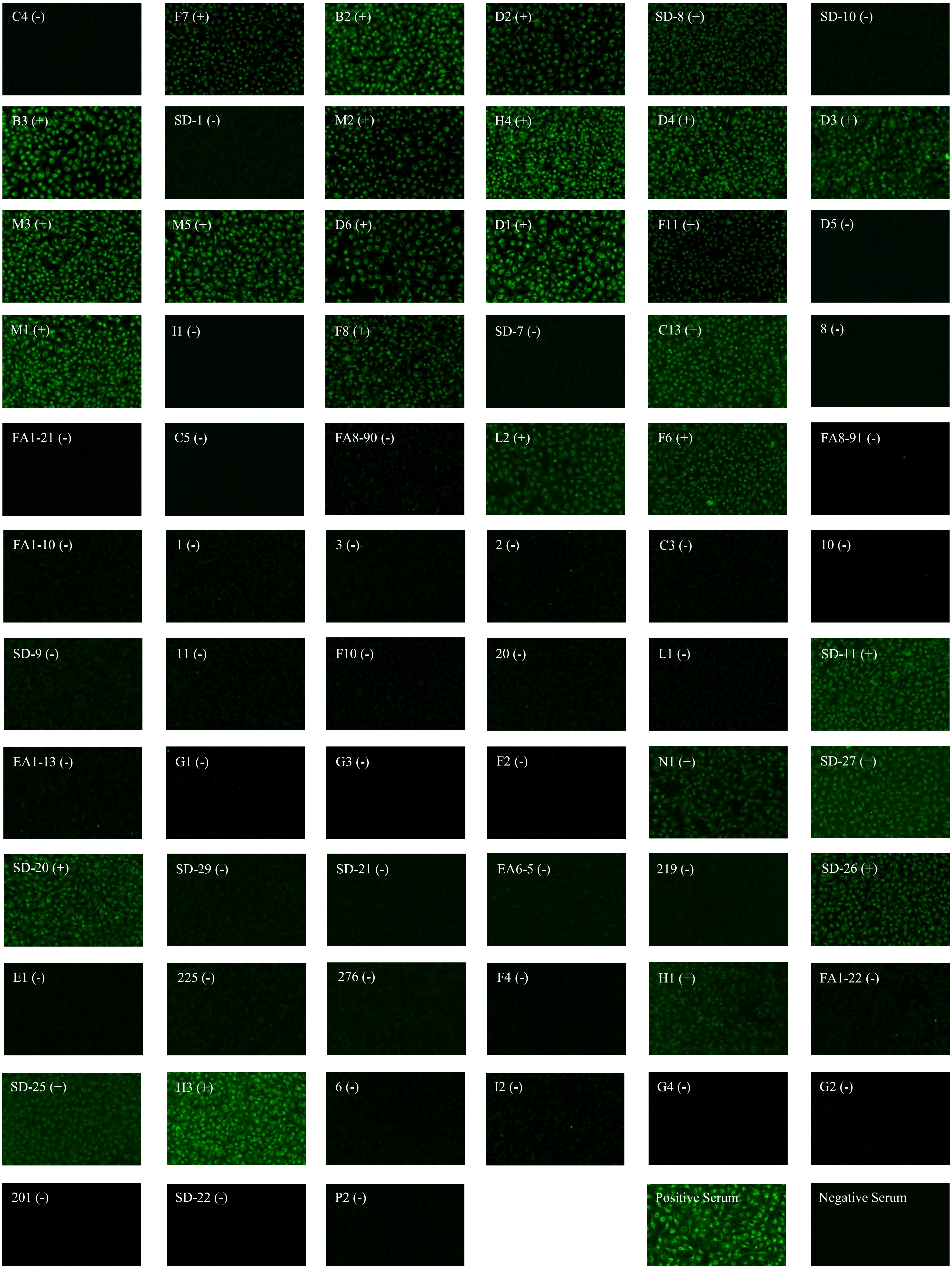
C4 (-)
F7 (+)
B2 (+)
D2 (+)
SD-8 (+)
SD-10 (-)
B3 (+)
SD-1 (-)
M2 (+)
H4 (+)
D4 (+)
D3 (+)
M3 (+)
M5 (+)
D6 (+)
D1 (+)
F11 (+)
D5 (-)
M1 (+)
I1 (-)
F8 (+)
SD-7 (-)
C13 (+)
8 (-)
FA1-21 (-)
C5 (-)
FA8-90 (-)
L2 (+)
F6 (+)
FA8-91 (-)
FA1-10 (-)
1 (-)
3 (-)
2 (-)
C3 (-)
10 (-)
SD-9 (-)
11 (-)
F10 (-)
20 (-)
L1 (-)
SD-11 (+)
EA1-13 (-)
G1 (-)
G3 (-)
F2 (-)
N1 (+)
SD-27 (+)
SD-20 (+)
SD-29 (-)
SD-21 (-)
EA6-5 (-)
219 (-)
SD-26 (+)
E1 (-)
225 (-)
276 (-)
F4 (-)
H1 (+)
FA1-22 (-)
SD-25 (+)
H3 (+)
6 (-)
I2 (-)
G4 (-)
G2 (-)
201 (-)
SD-22 (-)
P2 (-)
Positive Serum
Negative Serum

Fig. S1 Clinical serum samples were analyzed by IFA tests. The pig serum samples were diluted 1:500 in PBS, and incubated with GETV infected cells. The GETV positive or negative pig serum were used as control.
